# ﻿A new species and a newly recorded subgenus of *Lipotriches* Gerstaecker, 1858 (Hymenoptera, Apoidea, Halictidae, Nomiinae) from China

**DOI:** 10.3897/zookeys.1090.75872

**Published:** 2022-03-24

**Authors:** Dan Zhang, Ze-Qing Niu, Alain Pauly, Wa Da, Chao-Dong Zhu

**Affiliations:** 1 Key Laboratory of Zoological Systematics and Evolution, Institute of Zoology, Chinese Academy of Sciences, 1 Beichen West Road, Chaoyang District, Beijing, 100101, China; 2 College of Biological Sciences, University of Chinese Academy of Sciences, No.19A Yuquan Road, Shijingshan District, Beijing, 10049, China; 3 Royal Institute of Natural Sciences of Belgium, Entomology, Rue Vautier-29, B-1000 Brussels, Belgium; 4 Tibet Plateau Institute of Biology, Lhasa, 850001, Tibet, China; 5 State Key Laboratory of Integrated Pest Management, Institute of Zoology, Chinese Academy of Sciences, 1 Beichen West Road, Chaoyang District, Beijing, 100101, China

**Keywords:** Anthophila, Apiformes, bee pollinator, description, morphology, taxonomy

## Abstract

Two Chinese species of the genus *Lipotriches* Gerstaecker, 1858 are treated in this paper. Lipotriches (Lipotriches) guihongi Zhang & Niu, **sp. nov.** is recognized as a new species and Lipotriches (Maynenomia) nanensis (Cockerell, 1929) is a new species and subgenus record for China. The number of Chinese species of the subfamily Nomiinae and genus *Lipotriches* are updated to 47 and 15, respectively.

## ﻿Introduction

The genus *Lipotriches* (subfamily Nomiinae) was described by Gerstaecker (1858) with *Lipotrichesabdominalis* as the type species, including more than 340 species (Ascher and Pickering 2021). *Lipotriches* has a high diversity of species occurring throughout the Old World (Africa, Asia and Australia). Bee species of this genus are important pollinators for plants, especially for grasses (Pauly 2014a). Over the last few decades, various groups and species in this genus have been revised (Pauly 1984a, 2009, 2014a, b; Astafurova and Pesenko 2005; Michener 2007; Huang 2008; Niu et al. 2018). However, much more taxonomic work is needed for this group, especially in Asia.

In general, the body of *Lipotriches* species is relatively slender compared to most other nomiine species (Pauly 1990; Michener 2007). Morphological identification of the subgenus within *Lipotriches* strongly relies on adult characters: especially the pronotum with continuous, or medially, or laterally notched transverse carina in both sexes, and the basitibial plate of females with a carina only along the posterior margin (Michener 2007; Huang 2008). Pauly (1990) separated *Lipotriches* into several genera, such as *Austronomia* Michener, 1965, *Afronomia* Pauly, 1990, *Macronomia* Cockerell, 1917, and *Trinomia* Pauly, 1980. Later, Michener (2007) revised all the above groups as subgenera of the genus *Lipotriches*, dividing *Lipotriches* into nine subgenera in total.

Pauly (1984b) described *Maynenomia* as a genus with *Nomiamaynei* Cockerell, 1937 as the type species. Subsequently, he described nine new species and transferred five *Nomia* species to this group, giving a total of 15 species of *Maynenomia* in Africa and Asia (Pauly 2009). Michener (2007) treated *Maynenomia* as a subgenus within the genus *Lipotriches*, and suspected this subgenus probably to be a synonym of the subgenus Austronomia. While Pauly treated *Maynenomia* at genus level, based on its “oval” head shape, we follow Ascher and Pickering (2021) and Michener (2007) in treating *Maynenomia* as a subgenus within *Lipotriches*.

Herein, we reported two Chinese species of the genus *Lipotriches*, including one newly described species and one newly recorded species. To date, the Chinese species of subfamily Nomiinae is increased to 47 in total (Niu et al. 2018; Zhang et al. 2020).

## ﻿Materials and methods

In this study, a total of 50 specimens were examined, all of them were deposited in the Collection of the Institute of Zoology, Chinese Academy of Sciences, Beijing, China (**IZCAS**). The specimens were examined with Nikon SMZ 1500 stereomicroscope. Photographs were taken with Nikon D7000 digital camera and were stacked with Helicon Focus and Zerene Stacker. Final images were edited for clarity and mounted into plates by Photoshop CS6.

The morphological terminology follows Pesenko (1983) and Michener (2007) in this study. Absolute measurements were taken in millimeters (mm) for body length. The following abbreviations are used: BL, body length which was measured from basal antennal socket to the metasomal apex; HL, head length which represented the widest point of the head in frontal view; T1–5, the first to fifth metasomal terga; S1–8, the first to eighth metasomal sterna; F1–11, the first to eleventh flagellar segments. We measured the punctation density, punctation diameter (d) and the space between them (i), such as i = 1d or i<d.

## ﻿Taxonomy

**Genus *Lipotriches* Gerstaecker, 1858**

### ﻿Subgenus Lipotriches

Taxon classificationAnimaliaHymenopteraHalictidae

Gerstaecker

Lipotriches Gerstaecker, 1858: 460. Type species: Lipotrichesabdominalis Gerstaecker, 1857 = Sphecodescribrosa Spinola, 1843, monobasic.
Rhopalomelissa Alfken, 1926: 267. Type species: Rhopalomelissaxanthogaster Alfken, 1926, by designation of Sandhouse (1943: 596).
Nomia (Epinomia) Alfken, 1939: 113, not Ashmead, 1899. Type speies: Nomiaandrenoides Vachal, 1903 = Nomiaandrei Vachal, 1897, by original designation.
Alfkenomia Hirashima, 1956: 33, replacement for Epinomia Alfken, 1939. Type species: Nomiaandrenoides Vachal, 1903 = Nomiaandrei Vachal, 1897, autobasic.
Rhopalomelissa (Lepidorhopalomelissa) Wu, 1985: 58. Type species: Nomiaburmica Cockerell, 1920, by original designation.
Rhopalomelissa (Trichorhopalomelissa) Wu, 1985: 58. Type species: Rhopalomelissahainanensis Wu, 1985, by original designation.
Rhopalomelissa (Tropirhopalomelissa) Wu, 1985: 58. Type species: Rhopalomelissanigra Wu, 1985, by original designation.

#### Diagnosis.

Small-sized, body length about 5–12 mm; metasoma slender, with petiolate, T1 longer than broad in male (most species); pronotum with continuous or medially or laterally transverse carina or lamella anterior to the scutum; metasoma partly or wholly red for some species (i.e., *Lipotrichesfloralis*, *Lipotrichesesakii* and *Lipotrichesmediorufa*).

### ﻿ Lipotriches (Lipotriches) guihongi

Taxon classificationAnimaliaHymenopteraHalictidae

Zhang & Niu sp. nov.

http://zoobank.org/F11C65CF-98CB-4016-8AE6-4D101D75D5E8

Figs 1 , 2

#### Type material.

***Holotype***: China: 1♂, Xizang, Jilong County, Jilong Town, Jipu Village, 28°37'N, 85°32'E, 2744 m, 9 Aug. 2019, Dan Zhang, Qing-Tao Wu leg. ***Paratypes***: 5♀21♂, Jilong County, Jilong Town, Jipu Village, 28°37'N, 85°32'E, 2744m, 7–9 Aug. 2019, Dan Zhang, Qing-Tao Wu leg.; 16♀1♂, Xizang, Jilong County, Jilong Town, Xinjiang Village, 28°22'N, 85°21'E, 2727m, 6 Aug. 2019, Dan Zhang, Qing-Tao Wu leg.

#### Diagnosis.

**Figure 1.**
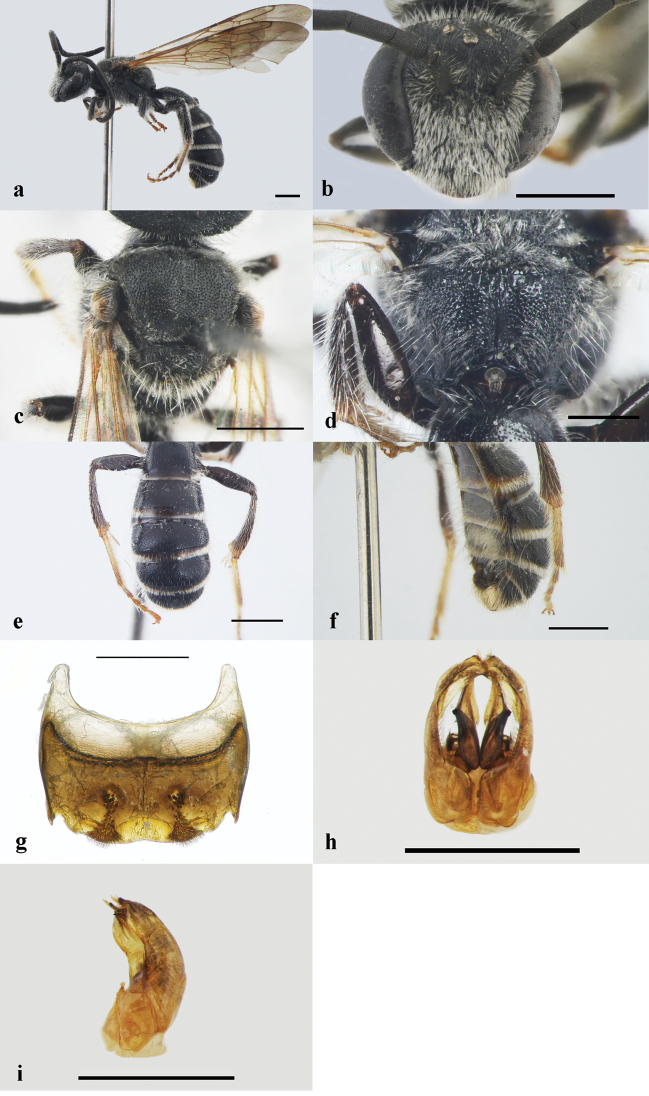
Lipotriches (Lipotriches) guihongi Zhang & Niu sp. nov., male **a** habitus in lateral view **b** head in frontal view **c** mesoscutum in dorsal view **d** propodeum in posterior view **e** metasoma in dorsal view **f** metasoma in lateral view **g** S5 in dorsal view **h** genitalia in dorsal view **i** genitalia in lateral view. Scale bars: 1 mm (**a–f**); 0.5mm (**g–i**).

Males of *L.guihongi* sp. nov. differ from other species of subgenus Lipotriches by the following combination of features: mesoscutum, metapostnotum and center disc of metasomal terga with dense and large punctures (Fig. 1c, d), S4 with dense short white hairs (Fig. 1f); S5 with a pair of circular protruding thickness on the disc, a pair of triangular feathery bristles connected on the apical margin (Fig. 1g); *L.guihongi* is most similar to *Lipotrichesyasumatsui* Hirashima, 1961 and *Lipotrichesceratina* (Smith,1857), however, the male of the new species can be distinguished from the two latter by a pair of large, dense, erect and brownish longitudinal tufted hairs on S5. In addition, S5 structure of *L.guihongi* sp. nov. is similar to *L.acanthospermi* Pauly, 2014b. We have found that the both species have dense tomentum on S4 and a pair triangular feathery bristles on the apical margin of S5, while the latter lacks a pair of circular protruding thickness on the disc of S5, and has only been found in Africa.

#### Description.

**Male** (measurements are only from the holotype). BL = 8 mm (Fig. 1a), body black. ***Head*.**HL: HW = 0.89, head broader than long (Fig. 1b); clypeus broader than long (Fig. 1b); paraocular area with dense large punctures (i = 0.1–0.2d; Fig. 1b); vertex with sparser large punctures (i = 0.5–1d; Fig. 1b); vertex behind shiny; posterior margin of vertex rounded (Fig. 1a, b); mandible blackish-brown; frons with dense and minute punctures, medial frontal line smooth (Fig. 1b); antenna reaching the posterior margin of T1; F1–11 almost equal in length, nearly 2.3 times as long as broad (Fig. 1a, b); ocelli normal (Fig. 1b). ***Mesosoma***. Mesoscutum, scutellum, and metanotum dull, without reflections (Fig. 1c). Mesoscutum with large and mostly confluent punctures (i = 0.2–0.3d; Fig. 1c); metanotum normal, without process, with large punctures which are sparser than punctures on the mesoscutum (Fig. 1c); posterior surface of propodeum with more larger and denser (i = 0.1–0.2d) punctures than those on the mesosoma (Fig. 1d); metapostnotum shiny, with broad longitudinal wrinkles (Fig. 1d); fore wing with three submarginal cells, the 1^st^ and 3^rd^ submarginal cell nearly equal in length, almost two times as long as 2^nd^ submarginal cells; tegula oval, yellow-brown, not enlarged (Fig. 1c); femur and tibia black (Fig. 1a); basitarsus, mediotarsus yellow-brown with tarsal claw (Fig. 1f); hind femur and tibia normal, not enlarged (Fig. 1c). ***Metasoma***. Surface of metasomal terga shiny, center of disc with dense punctures (i = 0.2–0.3d), apical of disc with sparser punctures than on the center (i = 0.5–1d); apical margin of T1–5 transparent (Fig. 1e, f); S5 with a pair of circular protruding thickness on the disc, a pair triangular feathery bristles on the apical margin connected (Fig. 1g); gonostylus as shown in Fig. 1h (in dorsal view) and Fig. 1i (in lateral view). ***Pubescence***. Clypeus, supraclypeal area and frons with white setae (Fig. 1a, b); scutellum with sparse long yellowish hairs (Fig. 1c); metanotum with dense pale tomentum (Fig. 1c); upper lateral surface of propodeum with sparse long white hairs (Fig. 1d); legs with sparse white hairs (Fig. 1a, d–f); apical area of metanotum with sparse long hairs (Fig. 1c); apical margin of T1–4 with white hair band, interrupted in middle (Fig. 1e); S4 covered with dense pale tomentum (Fig. 1f).

**Figure 2.**
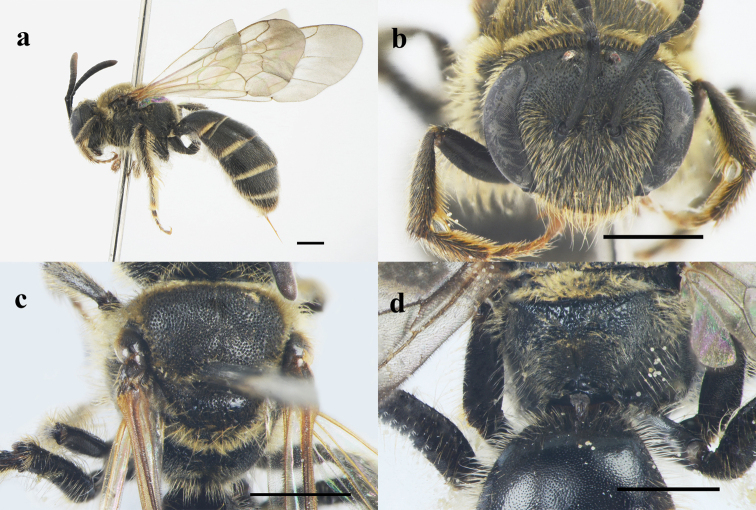
Lipotriches (Lipotriches) guihongi Zhang & Niu sp. nov., female **a** habitus in lateral view **b** head in frontal view **c** mesoscutum in dorsal view **d** propodeum in posterior view. Scale bars: 1 mm.

**Female.**BL = 10–12 mm. Similar to male, except the following: metapostnotum with punctures sparser (i = 0.4–0.5d) and smaller than male’s (Fig. 2d); head, mesosoma and metasoma with sparse yellowish hairs (Fig. 2b); T1–2 with densely and minutely punctures (i = 0.2–0.3d; Fig. 2d).

#### Etymology.

The name “*guihongi*” is dedicated to Prof. Hong Gui. He is a famous entomologist in China, who advised and encouraged Chao-Dong Zhu to continue his study on insects.

#### Floral association.

Unknown.

#### Distribution.

China (Xizang).

### Subgenus ﻿ Maynenomia

Taxon classificationAnimaliaHymenopteraHalictidae

Pauly, 1984

Maynenomia Pauly, 1984b: 698. Type species: Nomiamaynei Cockerell, 1937 = Nomiatestacea Friese, 1914, by original designation.

#### Diagnosis.

Glossa slender; apical of basitibial plate opened, delimited on both sides in female; scape short, not reaching median ocellus in females.

#### Distribution.

China (Yunnan); India (Uttarakhand); Indonesia; Laos; Malawi; Myanmar; South Africa; Tanzania; Thailand.

### ﻿ Lipotriches (Maynenomia) nanensis

Taxon classificationAnimaliaHymenopteraHalictidae

(Cockerell, 1929)

Fig. 3

Nomia nanensis Cockerell, 1929:133, ♀. Holotype, ♀, Thailande, Nan, USNM.
Maynenomia nanensis (Cockerell, 1929): Pauly 2009.

#### Material examined.

China: 2♀, Yunnan, Xishuangbanna, Naban River, 22°04'N, 100°22'E, 1303 m, 16 Jun. 2014, Xiu-wei Liu leg..

#### Diagnosis.

**Figure 3.**
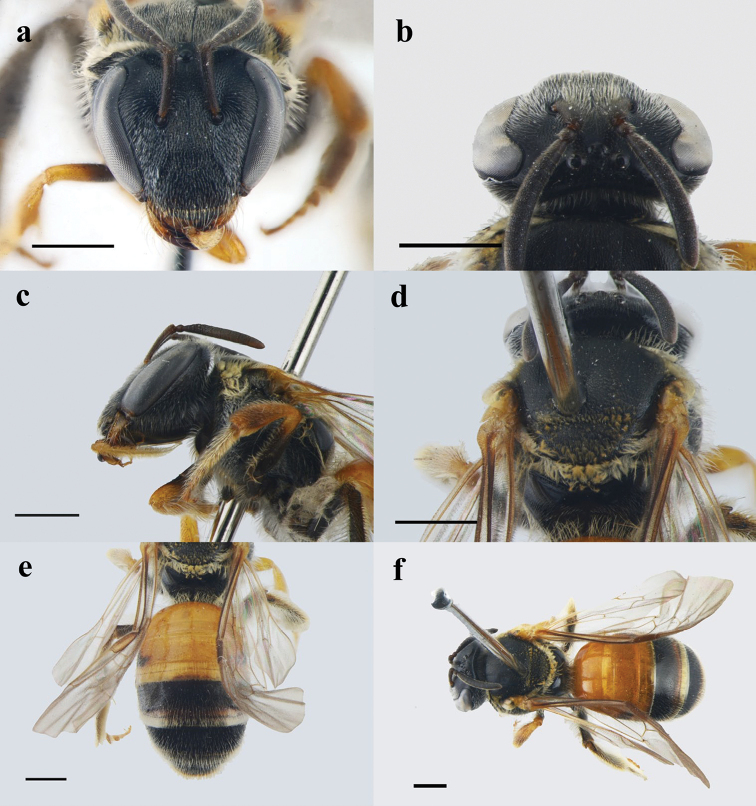
Lipotriches (Maynenomia) nanensis, female **a** head in frontal view **b** head in dorsal view **c** habitus in lateral view, showing the glossa **d** mesoscutum in dorsal view **e** metasoma in dorsal view **f** habitus in dorsal view. Scale bars: 1mm.

T1–2 mainly reddish, and T2 with large black spot at each side basally (Fig. 3e, f); legs reddish except fore femur black (Fig. 3c); mandibles subapically red, and apically black (Fig. 3c, f); hind tibia with basitibial plate (Fig. 3f); mesoscutum, scutellum and propodeum dull (Fig. 3d); mesoscutum with extremely dense and minute punctures (Fig. 3d); posterior surface of propodeum almost without punctures (Fig. 3d, e); metanotum with dense pale ochreous tomentum (Fig. 3d, e); surface of metasomal terga highly polished, with extremely sparse and minute punctures (Fig. 3e, f).

#### Distribution.

China (Yunnan); India (Maharashtra); Laos; Myanmar; Thailand.

#### Remark.

This species was recorded from China for the first time in this study, increasing the number of Chinese species of *Lipotriches* and Nomiinae to 15 and 47, respectively.

## Supplementary Material

XML Treatment for Lipotriches

XML Treatment for Lipotriches (Lipotriches) guihongi

XML Treatment for Maynenomia

XML Treatment for Lipotriches (Maynenomia) nanensis
